# Expanding Genetic Counselor Roles: A Model for Global Research Development

**DOI:** 10.3390/genes15070867

**Published:** 2024-07-01

**Authors:** Colleen C. Muraresku, Elizabeth M. McCormick, Lydia Rockart, T. Blaine Crowley, Stephanie Asher, Amanda Back, Sarah M. Baldino, Emma Bedoukian, Allison D. Britt, Natalie Burrill, Cara Cacioppo, Dana Farengo Clark, Mary Egan Clark, Laura Conway, Laynie Dratch, Holly A. Dubbs, Nicole M. Engelhardt, Natalie Ginn, Christopher Gray, Tiff Hartman, Evan R. Hathaway, Katherine L. Helbig, Lily Hoffman-Andrews, Stefanie Kasperski, Beth A. Keena, Kierstin N. Keller, Jessica M. Long, Lauren Lulis, Laina Lusk, Daniel E. McGinn, Rebecca Mueller, Rache A. Paul, Lisa Pilchman, Jacquelyn Powers, Sarah E. Raible, Sara Reichert, Alyssa L. Rippert, Angela G. Arnold, Sarah M. Ruggiero, Erica Schindewolf, Katie Rose Sullivan, Shannon Terek, Bekah Wang, McKenzie Wells, Natalia Wisniewski, Renee Wright, Elisabeth McCarty Wood, Stacy Woyciechowski, Kristin Zelley, Kathleen D. Valverde, Donna M. McDonald-McGinn

**Affiliations:** 1 Mitochondrial Medicine Frontier Program, Division of Human Genetics, Children’s Hospital of Philadelphia, Philadelphia, PA 19104, USA; mccormicke@chop.edu (E.M.M.); mcginnde@chop.edu (D.E.M.); 2 22q and You Center, Clinical Genetics, Division of Human Genetics, Children’s Hospital of Philadelphia, Philadelphia, PA 19104, USAmcginn@chop.edu (D.M.M.-M.); 3 Division of Translational Medicine and Human Genetics, Department of Medicine, Perelman School of Medicine, Penn Medicine, University of Pennsylvania, Philadelphia, PA 19104, USA; 4 Division of Neurology, Children’s Hospital of Philadelphia, Philadelphia, PA 19104, USAhelbigk@chop.edu (K.L.H.);; 5 Division of Neurology, The Epilepsy Neurogenetics Initiative, Children’s Hospital of Philadelphia, Philadelphia, PA 19104, USA; 6 Division of Oncology, Department of Pediatrics, Children’s Hospital of Philadelphia, Philadelphia, PA 19104, USAzelleyk@chop.edu (K.Z.); 7 Roberts Individualized Medical Genetics Center (RIMGC), Children’s Hospital of Philadelphia, Philadelphia, PA 19104, USA; 8 Division of Human Genetics, Children’s Hospital of Philadelphia, Philadelphia, PA 19104, USA; 9 Comprehensive Vascular Anomalies Program, Children’s Hospital of Philadelphia, Philadelphia, PA 19104, USA; 10 Richard D. Wood Jr. Center for Fetal Diagnosis and Treatment, Children’s Hospital of Philadelphia, Philadelphia, PA 19104, USA; 11 Penn Telegenetics Program, University of Pennsylvania, Philadelphia, PA 19104, USA; 12 Division of Hematology and Oncology, Department of Medicine, University of Pennsylvania, Philadelphia, PA 19104, USA; 13 Master of Science in Genetic Counseling Program, Perelman School of Medicine, University of Pennsylvania, Philadelphia, PA 19104, USAkathleen.valverde@pennmedicine.upenn.edu (K.D.V.); 14 Department of Neurology, University of Pennsylvania, Philadelphia, PA 19104, USA; 15 Section of Biochemical Genetics, Division of Genetics, Children’s Hospital of Philadelphia, Philadelphia, PA 19104, USA; 16 Division of Cardiovascular Medicine, Department of Medicine, Perelman School of Medicine, University of Pennsylvania, Philadelphia, PA 19104, USA; 17 Center for Mitochondrial and Epigenomic Medicine, Department of Pathology, Children’s Hospital of Philadelphia, Philadelphia, PA 19104, USA; 18 Basser Center for BRCA, Perelman School of Medicine, University of Pennsylvania, Philadelphia, PA 19104, USA; jessica.long@pennmedicine.upenn.edu (J.M.L.);; 19 Division of Genomic Diagnostics, Department of Pathology, The Children’s Hospital of Philadelphia, Philadelphia, PA 19104, USA; 20 Masters Genetic Counseling Program, Department of Medical Ethics and Health Policy, Perelman School of Medicine, University of Pennsylvania, Philadelphia, PA 19104, USA; 21 Division of Cardiology, Children’s Hospital of Philadelphia, Philadelphia, PA 19104, USA; 22 Division of Neurology, The Epilepsy Neurogenetics Initiative, Center for Epilepsy and Neurodevelopmental Disorders (ENDD), Children’s Hospital of Philadelphia, Philadelphia, PA 19104, USA; 23 CHOP Precision Medicine Services, Children’s Hospital of Philadelphia, Philadelphia, PA 19104, USA; schindewolfe@chop.edu; 24 Center for Applied Genomics, Children’s Hospital of Philadelphia, Philadelphia, PA 19104, USA; 25 Obstetrics and Gynecology Reproductive Genetics, Perelman School of Medicine, University of Pennsylvania, Philadelphia, PA 19104, USA; 26 Department of Medicine, Perelman School of Medicine, University of Pennsylvania, Philadelphia, PA 19104, USA; 27 Department of Pediatrics, Perelman School of Medicine, University of Pennsylvania, Philadelphia, PA 19104, USA; 28 Department of Human Biology and Medical Genetics, Sapienza University, 00185 Rome, Italy

**Keywords:** genetic counselors, research, publications

## Abstract

Purpose: Genetic counselors (GCs) increasingly play key roles in advancing genomic medicine through innovative research. Here, we examine one large cohort of GCs’ evolving contributions to the literature, with the goal of facilitating worldwide professional development for GCs through scholarly activities. Methods: Publications were cataloged by members of the Section of Genetic Counseling (Section), established at the Children’s Hospital of Philadelphia and the University of Pennsylvania in 2014, including publication year, journal, impact factor, and author position. Data were organized using the “My Bibliography” tool on the National Center for Biotechnology Information website and a Research Electronic Data Capture database created to initially collect manuscripts published through 30 June 2020. A subsequent survey captured publications through 5 February 2024. Results: An amount of 52 of 120 (43%) GCs shared their curriculum vitae/papers. 992 unique publications were identified from 1986 to 2024. Since 2013, no less than 32 papers were published annually by Section members and no less than 10 GCs contributed to publications yearly. Impact factors typically averaged >5.0 per year. Areas of foci diversified considerably since 2015. Conclusions: Here, we establish that GCs indeed contribute to scholarly work as evidenced by the number of publications alone. The establishment of an academic home may have contributed, given publications increased concurrent to launching the Section, providing a model for organizing GCs at institutions nationally and internationally. Highlighting such achievements will foster the expansion of GC roles in the era of precision genomic medicine and therapy. Considering ways to support GCs towards expanding these activities is equally important.

## 1. Introduction

Genetic counseling is the process of sharing information about and addressing the psychosocial implications of conditions with a genetic etiology with individuals at risk of developing or being personally affected by these conditions [1]. The genetic counseling process began in the United States (US) over 50 years ago and has been an international profession since the early 1990s. There are now 28 countries worldwide with practicing genetic or genomics counselors. At least 16 countries have master’s level training programs for genetic counselors (GCs) and at least 12 countries have formal regulation (a certification or registration body) for the profession [2].

In the US, more than half of GCs’ current work is in predominantly clinical or patient-facing roles, providing care for patients and families in the prenatal setting, hereditary cancer predisposition programs, adult genetics centers, pediatric genetic centers, specialty clinics, teaching, or in laboratory roles [2,3,4]. Other GCs in the US have expanded into industry, patient advocacy, non-academic institutions, and private practice environments. Additionally, some GCs have further evolved into other roles such as a genomic scientist in a clinical diagnostic or research laboratory, scientific writer, advisor, and/or leader in operations [4,5,6].

GCs are uniquely poised to bring their combined expertise in mechanisms and psychosocial implications of disease, as well as honed written and oral communication skills, to further research related to rare diseases. Indeed, in parallel to the growth of personalized genomic medicine worldwide, the intersection between clinical and research GC roles is primed for expansion in areas such as advancing the field as part of patient care, education around novel therapeutics, natural history studies, diagnostic interpretation of complex results, and clinical trials [7].

The Master of Science in Genetic Counseling (MSGC) training programs in the US have historically prioritized introducing an understanding of research practices and skillsets in students, including generating hypotheses and completing research studies. In addition, students manage large patient/research case files and data organization and master communication skills critical to both peer and patient/subject interactions, including the informed consent process, while ensuring a thorough understanding of ethical and practical challenges that arise within human subjects research [7]. In 2023, the Accreditation Council for Genetic Counseling (ACGC) Practice-based Competency for training GCs shifted the emphasis on research to include interpreting data and the literature; applying data and the literature considering its strengths, weaknesses, and limitations; and demonstrating knowledge of how genetic counselors engage and contribute to the research process.

In the US, the terminal degree for GCs remains a Master’s degree, which can limit opportunities for career advancement in a research setting. Indeed, in a 2003 US-based study, almost 85% of GCs reported participating in research in areas including case reports, natural history studies, and gene discovery; however, only ~35% reported anticipating continuing to work in the field in 10 years, with the most common reason for leaving being lack of room for advancement. Since then, the proportion of GCs self-reporting research roles in the US has varied, but in 2023, only 18% (518/2708) of GCs reported working in research [4].

GCs work daily in various settings: clinical, academic, industry, and others. There is also robust organization of GCs on the national level, such as the National Society of Genetic Counselors (NSGC) and the American Board of Genetic Counseling (ABGC). On a global level, GCs organized the Transnational Alliance for Genetic Counseling (TAGC), which was formed in 2008 to foster communication and collaboration among GCs from more than 20 countries. Organization on the local level in the US is more varied; however, there are regional and institutional commonalities in barriers and benefits that groups of GCs can leverage.

**Figure 1:**
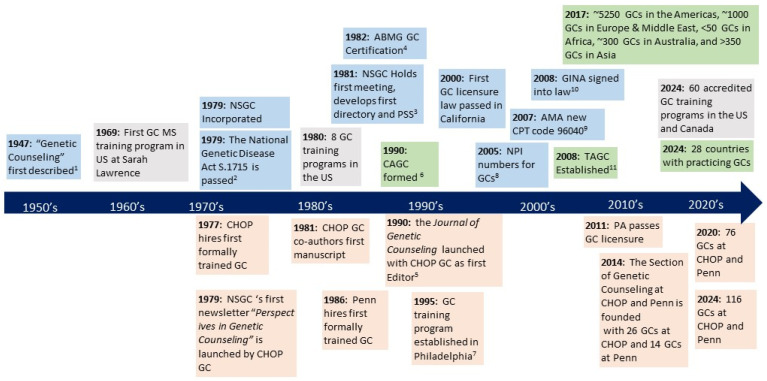
Timeline of the field of genetic counseling growth locally, nationally, and internationally.

The Children’s Hospital of Philadelphia (CHOP) and the University of Pennsylvania (Penn) organized the Section of Genetic Counseling (Section), established in 2014 (Figure 1). CHOP and Penn are university-based medical centers in close proximity geographically. CHOP specializes in pediatrics and Penn in adult medicine. Both fall under the umbrella of the Perelman School of Medicine at Penn, as does the Penn MSGC training program. Faculty appointments at CHOP and Penn are granted through Penn.

Employment of GCs has exploded across CHOP and Penn within the last several decades, as there was 1 GC at CHOP in the 1970s, 3 at CHOP and Penn in the 1980s, 40 in 2013, and 116 across the 2 institutions in 2024. The Section provides an academic home for GCs at CHOP and Penn regardless of the home department or division. The Section is driven by committees supporting clinical, educational, wellness, outreach, research activities, and professional development. The Section leads GC-related initiatives, ensures compliance with billing and licensure, encourages awareness of the profession by non-genetics professionals and the general public, organizes educational opportunities for GCs, GC students, GC assistants, undergraduates, and high school students, provides guidance in writing theses, abstracts, and manuscripts, and the development of IRBs and grants, while supporting the overall well-being of members by organizing regular social functions and wellness opportunities. In addition, the Section is closely aligned with the MSGC training program, with members acting as course directors, lecturers, committee members, clinical supervisors, and thesis advisors, with the latter lending itself nicely to supporting the growth of GCs in a research role.

(1) Sheldon Reed coined the term “genetic counseling” to describe the interaction of explaining the likelihood of passing a certain trait to offspring in 1947.
(2) The National Genetic Disease Act S.1715 of 1979 expanded funding for genetic counseling and broadened job opportunities for GCs across the USA.
(3) In 1981, the NSGC Professional Status Survey (PSS) of Full Members provided the first clinical, professional, educational, and demographic data for 238 genetic counselors. The first NSGC meeting was held in San Diego with 182 attendees supported by the March of Dimes. The first NSGC membership directory was published, listing 282 GCs from 35 states and 3 countries.
(4) In 1982, the American Board of Medical Genetics (ABMG) offered the first certification exam to GCs. This is the same general exam taken by medical and laboratory geneticists.
(5) The *Journal of Genetic Counseling* was launched in 1990 with an emphasis on research in genetic counseling with a CHOP GC as the first Editor.
(6) The Canadian Association of Genetic Counselors (CAGC) was formed in 1990.
(7) In collaboration with CHOP-Penn, Beaver College (later Arcadia University) established the first GC training program in Philadelphia, Pennsylvania (PA) in 1995.
(8) In 2005, the Centers for Medicare & Medicaid Services granted eligibility for genetic counselors to apply for NPI numbers.
(9) The American Medical Association (AMA) added a new CPT code 96040 for services provided by board-certified GCs in 2007.
(10) GINA (Genetic Information Nondiscrimination Act) was signed into law by US President George W. Bush in 2008.
(11) The Transnational Alliance for Genetic Counseling (TAGC) was established, including 19 countries to foster communication and collaboration among the international GC community and enhance genetic counseling education transnationally in 2008.

Region/Topic of GC activity: blue = nationally; grey = training; green = internationally;

orange = locally (CHOP/Penn)

With the global growth of the GC profession in recent years, it is important to understand the value of GC research. As more GCs are trained, genetic counseling services are expanding, and genetic testing is less expensive, accessible, and employed in numerous areas of medicine, the growth of research conducted by GCs is inevitable worldwide. This trend is evident in our academic institutions. The value GC research brings can be leveraged in academia, patient advocacy, and industry across the globe [2].

## 2. Materials and Methods

In 2020, GCs from the Section of Genetic Counseling (Section) at CHOP and Penn were provided instructions to upload their publications via the “My Bibliography” tool on the National Center for Biotechnology Information (NCBI) website to share with the Section’s Research Committee. Information including year of publication, journal name, current journal impact factor, author contribution (first author, senior, co-first, co-last, etc.), and publication type (gene discovery/diagnostics, phenotype expansion/case reports, clinical trials, psychosocial/genetic counseling issues, reviews/chapters, technology/innovation/new tools, genetic counseling career/supervising/programs, and other) was entered into a Research Electronic Data Capture (REDCap) database designed for this purpose.

A follow-up survey allowing GCs to update this information directly was developed in 2024 in REDCap and distributed to current Section members. Responses were collected and reviewed, unique PMIDs were captured where applicable, and responses were aggregated by item.

## 3. Results

**Figure 2:**
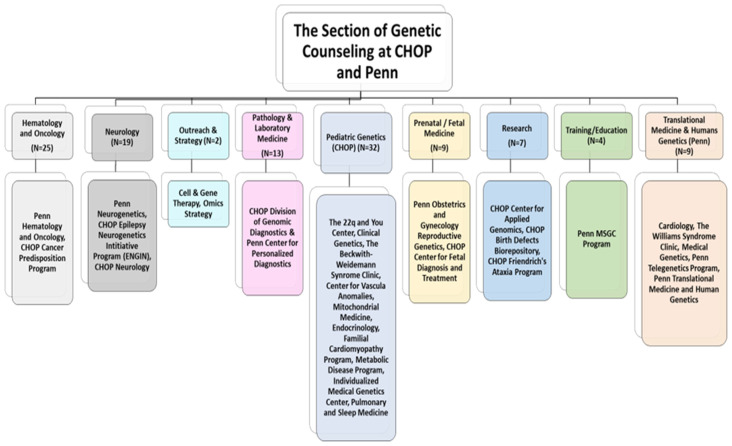
Genetic counselors’ divisions and specialties across CHOP and Penn. Genetic counselors and their divisions and specialized programs across the 2 institutions.

**Figure 3:**
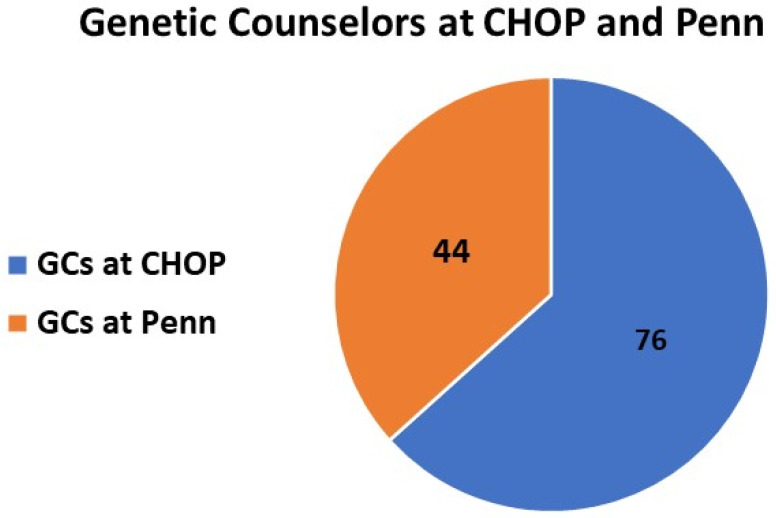
Genetic counselors in each institution in 2024. This represents the total number of individual genetic counselors at each institution, including the 4 who left, and who we have data on.

The Section currently includes 116 GCs. Four GCs who had entered data in 2020 have subsequently transferred employment outside of the Section, so their data was included in the analysis for a total of publication information from 120 CHOP-Penn GCs. A total of 37% (44/120) of GCs in the Section are employed at Penn and 63% (76/120) at CHOP (Figure 2). GCs from both institutions are supported across 9 divisions, including Neurology, Pediatric Genetics, Pathology and Laboratory Medicine, Hematology and Oncology, Research, Training/Education, Prenatal Genetic Diagnosis/Fetal Medicine, Outreach and Strategy, Translation Medicine, and Human Genetics. GCs are embedded in 30 programs across the Penn community, including the Division of Human Genetics at CHOP (Clinical Genetics Center, Biochemical Genetics, Individualized Medical Genetics Center, Mitochondrial Medicine, 22q and You Center, Beckwith–Wiedemann syndrome, William syndrome, Connective Tissue Program, Congenital Disorders of Glycosylation); Neurology at CHOP (Friedreich Ataxia Program, Neurogenetics, Genetic Epilepsy Disorders, Neuromuscular Disorders, and Leukodystrophy); other medical specialties at CHOP (Cardiology, Endocrinology, Vascular Anomalies, and Pulmonology); Hereditary Cancer Predisposition at CHOP and Penn including a robust telegenetics program at Penn; Prenatal Diagnostics and Fetal Medicine at CHOP and Penn; Outreach and Strategy (Omics Strategy and Cell and Gene Therapy); Translational Medicine and Human Genetics at Penn; Clinical Diagnostic Laboratory-based GCs housed in Pathology at CHOP and Penn; research-based roles (Translational Medicine at Penn, Genomics at CHOP including the Biorepository); and GC training (Penn MSGC Training Program) (Figure 3).

Thirty GCs shared their individual NCBI bibliography link in 2020. In total, 4 of the original 30 left the institution prior to 2024, so they were not invited to participate in the follow-up survey. One hundred and sixteen current Section members were invited to participate in the 2024 follow-up survey via email. Thirty-six responses were received, including from 14 GCs who previously shared their NCBI bibliography links in 2020.

**Figure 4:**
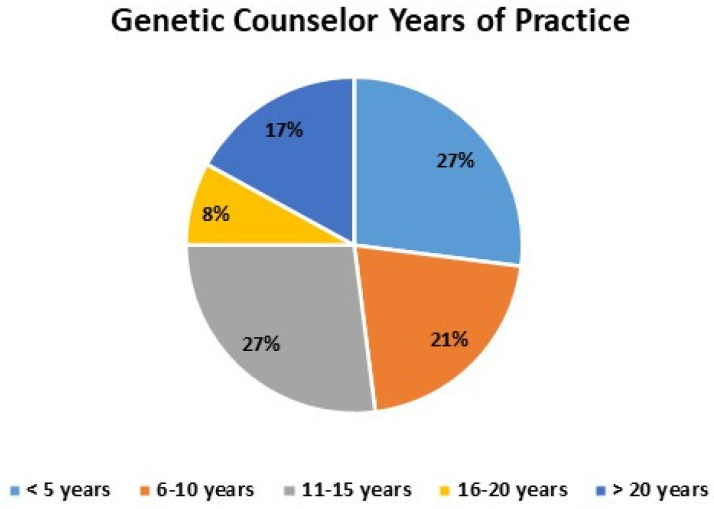
Numbers of unique publications per year and number of GC authors that year. Number of publications in the medical literature with at least one genetic counselor included as an author. With minimum increase in GCs publishing the number of publications increased some years 3-fold. 2024 data collected stopped as of 5 February.

In total, data were available from 52 GCs with a response rate of 43% (52/120), 67% (35/52) from CHOP and 33% (17/52) from Penn. In total, 27% (14/52) were practicing GCs for less than 5 years, 21% (11/52) had 6–10 years experience, 27% (14/52) had 11–15 years experience, 8% (4/52) had 16–20 years experience, and 17% (9/52) had greater than 20 years experience (Figure 4).

**Figure 5:**
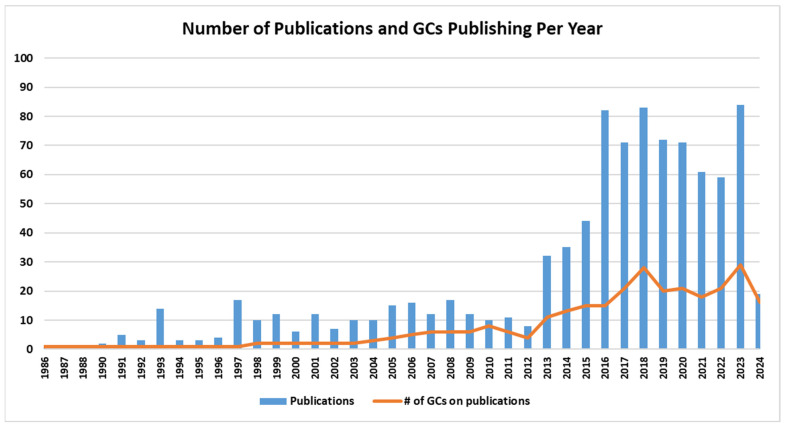
Numbers of unique publications per year and number of GC authors that year. Number of publications in the medical literature with at least one genetic counselor included as an author. With minimum increase in GCs publishing the number of publications increased some years 3 fold. 2024 data collected stopped as of 5 February.

**Figure 6:**
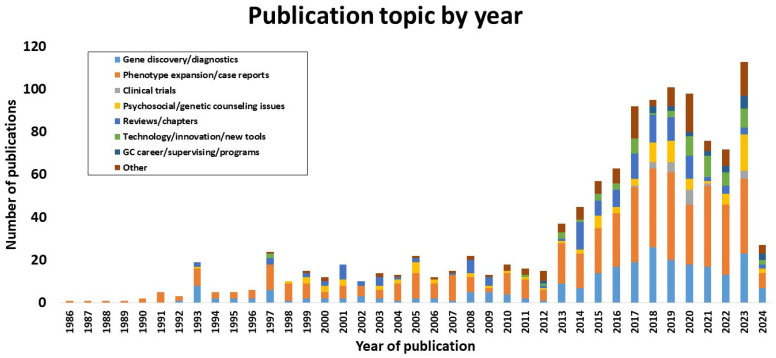
Categories of publications per year. Genetic counselors contributed to diverse publication types. 2024 data collected stopped as of 5 February.

GCs contributed to almost 1000 publications in the scientific literature across a 38-year span (1986–2024; Figure 5), with 9% (89/992) of manuscripts published in 2023 and 19 accepted for publication from 1 January to 5 February 2024. Of note, the number of publications increased considerably after 2013 as no less than 32 manuscripts were published yearly, culminating with a maximum of 89 publications in 2023. When analyzing this against the number of GCs who were active in research, impressively, in most years post-2012, the number of publications was triple the amount of GC authors. These data show that, in 2023, from our cohort, 56% (29/52) of GCs who responded published that year. This has been steadily above 35% (18/52) of respondents since 2017. The impact factor varied annually but typically averaged >5.0. One hundred and eighty-four (19%) papers were published in journals with impact factors >10.0, 24 (2%) were published in journals with impact factors of >20, and 11 (1%) were published in journals with impact factors of >30. Similarly, areas of focus gradually became more diverse, although expansion of phenotypes/case reports was the most common topic overall. However, since 2015, a shift towards gene discovery/diagnostics, clinical trials, and reviews has been observed (Figure 6).

**Figure 7:**
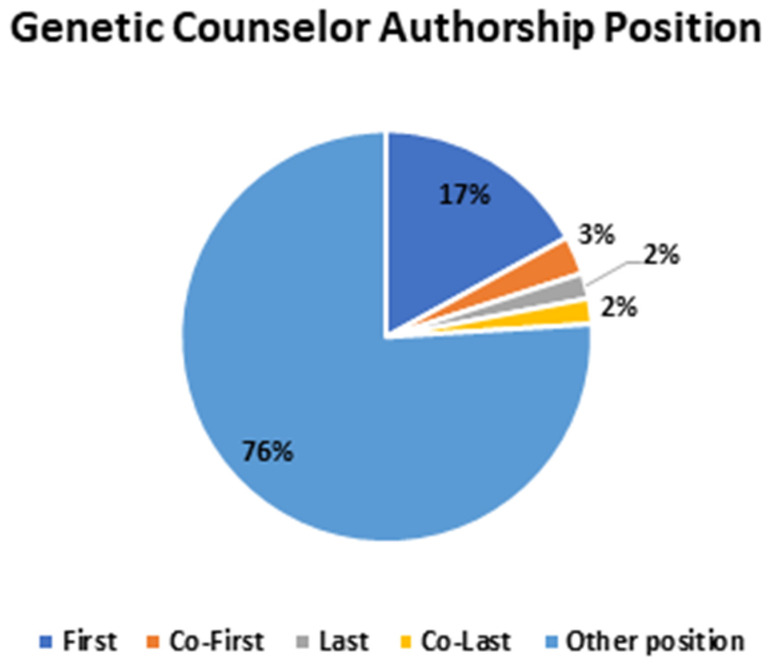
Genetic counseling authorship position. Genetic counselors held various positions in authorships across the medical literature.

In a review of authorship position, 24% (242/992) of publications included a GC in a position indicating significant contribution, such as 17% (172/992) as first authors, 3% (26/992) as co-first, 2% (21/992) as last authors, and 2% (23/992) as co-last authors (Figure 7).

## 4. Discussion

Of the 6517 certified GCs in the US in 2023 [4], 116, or roughly 2%, are current members of the Section of Genetic Counseling at CHOP and Penn. Here, we report on a subset of responders’ contributions to the scientific literature, which may well provide a paradigm for GCs’ participation in research not only in academia but across a variety of settings globally. Our data support the assertion that GCs have pivotal roles in manuscript preparation and publication, as both collaborators and primary authors, contributing to the scientific literature on a wide range of topics related to genomic medicine and precision therapy. Not surprisingly, GCs most commonly publish case reports centered around gene discovery and phenotype expansion. However, they also actively contribute to reports on clinical trials and technology and are regularly invited authors for topic reviews and diagnosis-specific healthcare guidelines. GC contributions are essential in papers that are widely read and highly cited, as evidenced by the high impact factors for the journals in which they publish. Moreover, the critical role of GCs in research activities is evident in their roles as first and/or senior authors of many of these important publications.

In recognition of the strong research interests and contributions to research among the CHOP and Penn GCs, the Master of Science in Genetic Counseling Program at the Perelman School of Medicine of the University of Pennsylvania established the Advanced Research Training for Genetic Counselors (ART-GC) certificate program in March of 2023. The ART-GC is a post-masters graduate level program that provides GCs with specific advanced training in research to prepare them to lead biomedical, translational, behavioral and/or clinical research studies in genomic medicine. It is a part-time online one-year program that combines four didactic courses and mentored research experience. The first cohort of ten GCs began the program in August 2023. The students in the ART-GC program are funded by a generous grant from the Warren Alpert Foundation, the Career Ladder Education Program for GCs that supports 50% of their salary to have protected, dedicated time to advance their research projects and covers the cost of all tuition and fees. This program will certainly support the further development of careers in research for GCs in Philadelphia, across the US, and hopefully around the world, as the Penn MSGC program and Section lead are currently in discussions to enhance training for those interested in pursuing a career in GC in Santiago, Chile, and more broadly across South America with opportunities for online education and binational clinical placements.

Despite the clear contributions of GCs to research, ACGC competencies have changed for research in the GC education setting in the US. This is consistent globally as there are limited programs offering research training opportunities. This raises concern for the “brain-drain” from GCs envisioning themselves leaving the field over time given the dearth of opportunities for academic advancement. Indeed, there has been a steady decline of GCs employed in academic institutions—60% in 1980 to 37% (1052/2708) in 2023—undoubtedly related in part to the expansion of GC roles in other areas such as clinical diagnostic laboratories; however, it is also likely related to a paucity of professional development opportunity [8]. In the US, GCs play a critical role in research and discovery, evidenced by the significant contributions to the scientific literature presented in this review.

Organizing GCs at CHOP and Penn within the Section has allowed for focused attention and a better understanding of GC roles, including research-related roles. Identifying GC roles in research, particularly as contributing authors to the scientific literature, is a step towards understanding the current and future academic strengths of a subset of genetics professionals waiting for an opportunity to launch themselves, whether into a primary research career or growing professionally to include research activities into their role, but not in a laboratory setting nor psychological domain alone. GCs combine unique interests and skill sets in basic science and psychosocial investigations, often concentrating on distinct areas of interest, some epidemiologic, others disease-specific, but often unexplored. This also provides unique opportunities to diversify GC funding outside of the traditional healthcare model where funding can be limited worldwide, as well as billing and collection rates remain low [9]. Indeed, GC roles can be partially or fully supported under a research umbrella by a variety of funding mechanisms including federal and local grants, philanthropy, and industry. 

Recognizing the barriers worldwide in integrating GCs into the workforce could help expand professional roles. Having GCs engaged in clinical research can aid clinical and research collaborations, support mentoring trainees and current and future colleagues, and continue to contribute to the scientific literature with the goal of facilitating mutual opportunities for innovation, discovery, and treatment.

In summary, here, we establish that GCs indeed contribute to scholarly works, as evident by the sheer number of publications alone, but also by the impressive impact factors, the position of GCs as authors on many publications, the types of publications, and by the fact that a funding agency (the Warren Alpert Foundation) has considered research training for GC a priority by supporting the establishment of a mechanism to train GCs specifically to perform scientific inquiries. It seems that the establishment of an academic home may have contributed to the success of the GCs at CHOP and Penn, given that the publications increased concurrently with the launching of the Section. Thus, the Section of Genetic Counseling at CHOP and Penn may well provide a model for organizing GCs at other institutions, both nationally and internationally, towards supporting practical activities, such as obtaining CEUs and systematizing clinical rotations, as well as professional development, in particular encouraging those with an interest in taking part in research activities to grow into genomic scholars. Highlighting such achievements to the international GC community will no doubt foster the expansion of GC roles in the era of precision genomic medicine and therapy.

Possible future directions include assessing how research-supported time intersects with clinical demands, as well as gaining an improved understanding of additional barriers GCs face to pursue research. Considering ways to support GCs towards expanding these activities, such as the development of a GC-specific PhD path, is equally important, as providing opportunities for GCs seeking higher education to prepare them for a research career allows GCs to be primary researchers to expand the genetic counseling literature worldwide.

## Data Availability

The original contributions presented in the study are included in the article, further inquiries can be directed to the corresponding author.
